# Benign lesions prolapsing through the anus: a differential diagnostic algorithm and evidence-based surgical management—a narrative review

**DOI:** 10.3389/fsurg.2026.1825956

**Published:** 2026-06-11

**Authors:** Jose Antonio Vergara Torrente, Luis Alfonso Huerta, Carlos Federico Sanchez, Pamela Valdez, Alondra Esparza González, Gerardo Muñoz Maldonado

**Affiliations:** Department of General Surgery, Hospital Universitario Dr. José Eleuterio González, Universidad Autónoma de Nuevo León, Monterrey, Nuevo León, Mexico

**Keywords:** anal canal polyps, anal prolapse, clinical algorithm, condyloma acuminatum, differential diagnosis, hemorrhoids, hypertrophied anal papilla, rectal prolapse

## Abstract

Masses prolapsing through the anus represent a frequent diagnostic challenge in coloproctological practice. Misidentification may delay the management of malignant disease or surgical emergencies. This narrative review integrates current evidence on seven benign entities that may prolapse through the anus—complete rectal prolapse, mucosal rectal prolapse, internal hemorrhoids, thrombosed hemorrhoids, anal condylomas, hypertrophied anal papilla, and anal canal polyps—and proposes a clinical decision algorithm based on findings from physical examination. Particular emphasis is placed on the identification of red flags that require exclusion of malignancy or surgical emergency. The evidence supporting the different therapeutic options is critically analyzed. The proposed algorithm prioritizes objective clinical characteristics—fold pattern, texture, mobility, and anatomical location—to guide differential diagnosis and the sequence of complementary studies, optimizing surgical decision-making.

## 1 Introduction

Benign prolapsing anal lesions represent a common reason for consultation in coloproctology, with prevalences ranging from 0.5% for rectal prolapse to 33% for condylomas in high-risk populations. Despite their predominantly benign nature, inadequate differential diagnosis may result in three clinically relevant scenarios: delay in the identification of malignant disease (anal squamous cell carcinoma, rectal adenocarcinoma, or leiomyosarcoma), suboptimal management of surgical emergencies (strangulated prolapse or hemorrhoidal thrombosis), and unnecessary or ineffective treatments due to diagnostic errors.

**Figure 1:**
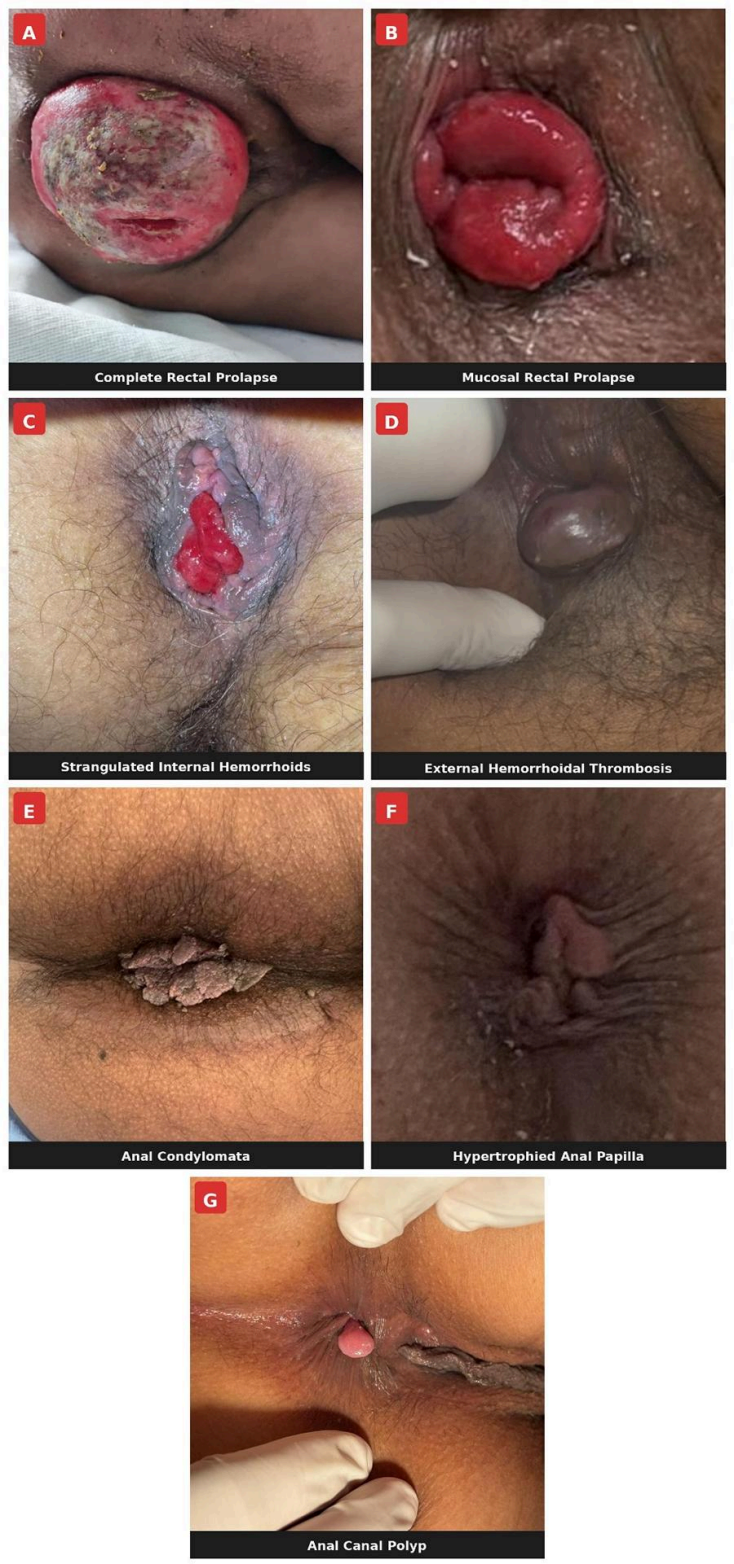
Clinical images of the most frequent benign lesions prolapsing through the anus. (**A**) Complete rectal prolapse. (**B**) Mucosal rectal prolapse. (**C**) Strangulated internal hemorrhoids. (**D**) External hemorrhoidal thrombosis. (**E**) Anal condylomata. (**F**) Hypertrophied anal papilla. (**G**) Anal canal polyp.

Differential diagnosis among these entities is primarily based on directed physical examination and lesion characteristics such as fold pattern, number of lesions, mobility, texture, predominant symptom, and comorbidities (Table 1). However, the literature lacks operational algorithms that integrate objective clinical findings into validated decision sequences. This review synthesizes current evidence on the benign entities most frequently presenting with prolapse (Figure 1), with emphasis on clinical differentiation, identification of red flags, and therapeutic stratification according to level of evidence.

**Table 1:** Clinical differential diagnosis of prolapsing anal lesions.

Characteristic	Rectal prolapse	Mucosal rectal prolapse	Internal hemorrhoids	Thrombosed hemorrhoids	Anal condyloma	Hypertrophied anal papilla	Anal polyp
Fold pattern	Circumferential concentric	Radial or mucosal ring	Radial folds, segmental masses (3–4 columns)	Absent folds (single nodule)	Absent (verrucous surface)	Single pedunculated	Pedunculated
Surface texture	Smooth mucosa	Smooth edematous mucosa (if prolapsed)	Congestive edematous pink purple mucosa	Violaceous tense edematous	Multilobulated verrucous	Firm smooth	Variable
Mobility	Reducible (if not strangulated)	Reducible	Reducible (grade III) or irreducible (grade IV)	Fixed not reducible	Fixed to skin or mucosa	Mobile on pedicle	Mobile on pedicle
Location	Circumferential 360°	Distal anal canal, mucosal ring at dentate line	Anal canal, dentate line, typical columns	External anal margin (subcutaneous)	Perianal ± intraanal	Posterior dentate line	Anal canal 0–2 cm above dentate line
Number of lesions	Single	Single	Single or Multiple	Single or multiple	Multiple (>80%)	Single (associated with fissure)	Single or multiple
Pain	No (except if strangulated)	No (mild discomfort only)	No (except grade IV strangulated)	Intense, constant	No (pruritus only)	No (only if >2 cm)	No
Associated finding	Fecal incontinence (41%–83%)	Soiling, hemorrhoidal coexistence	Bleeding, mucorrhea	Sudden onset (<24 h)	Other sexually transmitted infections (40%–60%)	Chronic anal fissure (>90%)	Mild bleeding
Predominant age	>60 years	Variable	Variable	30–50 years	20–40 years	Variable	Variable
Sex predominance	Female 6:1	No predominance	No predominance	No predominance	Male (if MSM)	No predominance	No predominance

STI, sexually transmitted infections; MSM, men who have sex with men.

Although external hemorrhoidal thrombosis does not prolapse through the anal canal in the strict anatomical sense, it is included as a relevant differential diagnosis given its frequent clinical overlap with truly prolapsing lesions, its presentation in the same anatomical region, and its potential for misidentification during physical examination.

Similarly, anal condylomas are included despite rarely presenting as true prolapsing lesions in the strict anatomical sense. Their inclusion is justified by their frequent occurrence in the perianal region, their clinical overlap with other prolapsing lesions during physical examination, and their importance in the differential diagnosis of exophytic perianal masses.

### 1.1 Objectives

1. To characterize the distinctive clinical findings of each entity.
2. To identify red flags that require exclusion of malignant or urgent pathology.
3. To provide a diagnostic algorithm based on physical examination.
4. To stratify therapeutic options according to quality of evidence.

## 2 Methods

This narrative review synthesized the current evidence on benign prolapsing anal lesions through a search in PubMed, Embase, and the Cochrane Library (January 2014 to December 2024). The search strategy used MeSH terms—rectal prolapse, hemorrhoids, condyloma acuminata, anal papilla, and anorectal neoplasms—combined with the words diagnosis, surgery, and treatment. The full search string used was (“rectal prolapse”[MeSH] OR “hemorrhoids”[MeSH] OR “condyloma acuminata”[MeSH] OR “anal papilla”[tiab] OR “anorectal neoplasms”[MeSH]) AND (“diagnosis”[tiab] OR “surgery”[tiab] OR “treatment”[tiab] OR “management”[tiab]), applied across all three databases with appropriate field tags. No PRISMA flowchart was generated given the narrative design; however, the selection and synthesis process is detailed in the following to ensure methodological transparency.

Clinical practice guidelines from scientific societies [American Society of Colon and Rectal Surgeons (ASCRS), European Society of ColoProctology (ESCP), and SICCR], systematic reviews, meta-analyses, and comparative studies were prioritized. Levels of evidence were assigned according to the Oxford Centre for Evidence-Based Medicine classification.

Registration of a systematic review protocol was not performed due to the narrative nature of the synthesis. The search was limited to literature in English, Spanish, and Italian. Reference selection prioritized methodological quality over exhaustiveness, with emphasis on studies published within the last 10 years to reflect contemporary practice.

### 2.1 Inclusion and exclusion criteria

Studies evaluating diagnosis, treatment, or prognosis of benign prolapsing anal lesions in adult populations were included. Eligible designs comprised randomized clinical trials, systematic reviews, meta-analyses, prospective or retrospective cohort studies, and clinical practice guidelines from coloproctology societies. Isolated case reports without literature review, editorials without original data, and studies exclusively in pediatric populations less than 18 years of age were excluded. For entities with limited high-quality evidence (hypertrophied anal papilla and anal canal polyps), case series with *n* ≥ 10 were accepted.

### 2.2 Selection and synthesis process

The initial search identified 1,847 records (PubMed: 1,124; Embase: 598; and Cochrane Library: 125). After removal of duplicates (*n* = 312), 1,535 titles and abstracts were screened, with 1,401 excluded for not meeting thematic or design criteria. A total of 134 full-text articles were assessed, of which 40 references were finally included corresponding to clinical practice guidelines (*n* = 8), systematic reviews and meta-analyses (*n* = 5), observational and cohort studies (*n* = 11), narrative and expert reviews (*n* = 13), and selected case series (*n* = 3). Data extraction was performed by the primary author, and disagreements were resolved by consensus. Levels of evidence were assigned according to the Oxford Centre for Evidence-Based Medicine classification (2011), with grades ranging from A (consistent Level I evidence) to D (Level V or inconsistent evidence).

## 3 Internal and thrombosed hemorrhoids

### 3.1 Definition and pathophysiology

Hemorrhoidal disease corresponds to the pathological distal displacement and congestion of the anal vascular cushions (superior and inferior hemorrhoidal plexuses), which are normal anatomical structures that contribute to fine continence through their engorgement at rest (1, 2). The pathogenesis is multifactorial: laxity of the subepithelial connective tissue, chronic vascular congestion, and mechanical trauma due to repeated straining (2).

External hemorrhoidal thrombosis (THE): This is defined as the sudden formation of an intravascular clot in the inferior hemorrhoidal plexus submucosal, below the dentate line, typically triggered by an abrupt increase in intraabdominal pressure (3). The thrombus produces acute distension of the fibrous capsule of the vascular cushion, resulting in severe pain disproportionate to the size of the lesion.

Strangulated internal hemorrhoids (SIHs): These occur when irreducible prolapse (grade IV according to Goligher) produces sustained contraction of the internal anal sphincter over the prolapsed hemorrhoidal bundles, compromising venous and lymphatic return. This leads to progressive edema, massive congestion, secondary thrombosis, and, if untreated, mucosal necrosis with risk of ulceration and superinfection (2, 4).

Current controversy: Although strangulation has traditionally been described as mechanical sphincter compression, anal pressure studies suggest that the predominant component is extensive venous thrombosis rather than arterial compression (2).

### 3.2 Epidemiology and risk factors

The global prevalence of symptomatic hemorrhoidal disease is estimated at 4%–5% in the general adult population, although endoscopic studies detect asymptomatic hemorrhoids in up to 40% of adults older than 50 years of age (5). Acute episodes of thrombosis (THE) account for 10%–15% of consultations in proctologic emergency settings, with peak incidence in the fourth and fifth decades of life (1, 6).

Modifiable risk factors:

- Chronic constipation with excessive straining (40%–60% of symptomatic cases) (4, 5).
- Chronic diarrhea causing repeated mechanical irritation.
- Sedentary lifestyle and obesity leading to pelvic venous congestion.
- Low-fiber diet (less than 20 g per day).

Non-modifiable factors:

- Pregnancy and childbirth: sustained increase in intraabdominal pressure.
- Advanced age: degeneration of supporting connective tissue.

Acute triggering factors:

- Intense defecatory effort.
- Sudden lifting of weights.
- Prolonged coughing or labor.

### 3.3 Clinical manifestations

#### 3.3.1 External hemorrhoidal thrombosis

THE typically presents with acute anal pain of sudden onset (less than 12–24 h), reaching maximal intensity during the first 48–72 h. The pain is constant and not pulsatile—distinguishing it from abscess—and is exacerbated by sitting, defecation, and direct pressure. Patients may report a bursting sensation during straining (3, 6).

Bleeding during THE is rare (less than 10%). Systemic symptoms such as fever or malaise suggest an infectious complication rather than simple thrombosis (7).

##### 3.3.1.1 Strangulated internal hemorrhoids

SIHs typically present with dull, constant pain accompanied by a sensation of rectal fullness and tenesmus. The condition develops gradually, progressing from previously reducible prolapse to one that becomes irreducible (4, 8). Bleeding may occur due to mucosal ulceration (10%–20%), and mucous discharge is frequent as a result of prolapsed mucosa.

The duration of symptoms is critical for therapeutic decision-making. Patients presenting more than 72 h after onset typically show spontaneous improvement and do not benefit from surgery (6, 9).

### 3.4 Diagnosis

Optimal examination is performed in the left lateral decubitus (Sims) position with knees flexed. Gentle separation of the buttocks allows inspection without initial painful digital rectal examination (1).

#### 3.4.1 External hemorrhoidal thrombosis

Pathognomonic findings:

- Nodule: single subcutaneous nodule, occasionally multiple, located at the external anal margin, any quadrant, most commonly posterior.
- Size: 0.5–3 cm; median 1.5 cm.
- Color: violaceous, bluish, or blackish if superficial skin necrosis is present.
- Overlying skin: tense, shiny, intact or ulcerated if necrosis is present.
- Consistency: firm, elastic on gentle palpation; fluctuance suggests evolution to abscess (rare, less than 2%).
- Borders: well-defined, mobile over the deep plane, not fixed to the sphincter.

### 3.5 Critical differential diagnosis

- Perianal abscess: fluctuance, diffuse erythema, local heat, and fever. THE does not present these signs.
- Thrombosed sentinel skin tag: associated with fissure, typical posterior midline location, with stabbing postdefecatory pain.
- Prolapsed fibroepithelial polyp: pedunculated, painless, and mobile.

### 3.6 Strangulated internal hemorrhoids

Pathognomonic findings:

- Mass: circumferential mass of prolapsed edematous mucosa occupying the entire anal canal and protruding through the anal orifice.
- Folds: radial folds characteristic of hemorrhoids, in contrast to concentric folds in complete rectal prolapse (7, 8).
- Color: congestive, dark red, or violaceous, with areas of ecchymosis, petechiae, or ulceration if necrosis is present.
- Consistency: edematous, soft, friable, with easy bleeding on contact.
- Irreducibility: mass cannot be reduced with gentle manual maneuver.
- Anal sulcus: absent between the mass and the perianal skin; prolapsed mucosa is continuous with the anoderm.

### 3.7 Clinical severity classification

- Mild: moderate edema, pink erythematous mucosa without ulceration, less than 48-h evolution.
- Moderate: severe edema, violaceous congestion, petechiae without frank necrosis, 48–72-h evolution.
- Severe: mucosal necrosis, black-grayish areas, deep ulceration, foul odor, signs of perianal cellulitis, more than 72-h evolution or immunocompromised patient.

Digital rectal examination: This exam must be performed with extreme caution, as pain may limit tolerance (3). It is indicated to rule out suspicious intraluminal rectal masses, evaluate baseline sphincter tone, and identify deep purulent collections. Rather than being considered absolutely contraindicated, digital rectal examination should only be performed under optimal conditions: adequate analgesia, patient cooperation, and controlled setting. In cases of extreme pain, suspicion of Fournier gangrene, or inability to ensure a gentle and controlled examination, it should be deferred until conditions are optimized.

Complementary studies: Acute hemorrhoidal disease is a clinical diagnosis. Complementary studies are not routine but may be directed when specific diagnostic doubts arise (1, 3, 8).

### 3.8 Treatment

The choice between conservative and surgical management is based on three objective criteria:

1. Time of evolution: Onset less than 72 h favors surgery; onset beyond 72 h favors conservative management (6, 9).
2. Clinical severity: Tissue necrosis, uncontrolled bleeding, or incapacitating pain despite analgesia requires surgery (10).
3. Patient preference: Patient can make an informed choice after explanation of risks and benefits.

Current controversy: ASCRS and ESCP guidelines recommend offering surgery in cases of THE within 72 h of onset, but acknowledge that the evidence is of moderate quality (Level II–III) and that conservative management is acceptable if the patient prefers to avoid surgery (3, 8). For SIHs, recent evidence favors early surgery, reducing recovery time and recurrence, but only in surgically fit patients (11).

#### 3.8.1 External Hemorrhoidal Thrombosis

##### 3.8.1.1 Conservative management: indications beyond 72 h or patient preference

Conservative treatment is considered first-line in cases presenting more than 72 h after onset or when the patient declines surgery. The objective symptomatic relief while the thrombus undergoes spontaneous reabsorption within 2–4 weeks (Level V, expert opinion) (6).

##### 3.8.1.2 Components of treatment

1. Systemic analgesia: nonsteroidal anti-inflammatory drugs (NSAIDs)—ibuprofen 400–600 mg every 8 hours or naproxen 500 mg every 12 hours—as first-line therapy. Paracetamol 1 g every 8 hours if NSAIDs are contraindicated. Opioids should be avoided because constipation worsens symptoms.
2. Local measures: Measures include warm sitz baths (38–40°C) for 10–15 min, performed three to four times per day (Level IV: case series) (3, 6). Application of local cold for 10–15 min every 2–4 h during the first 48 h may also provide relief. In addition, topical creams or ointments such as lidocaine 2%–5% or hydrocortisone 1% can be used (Level III).
3. Flavonoid therapy: Purified micronized flavonoid fraction is given at a loading dose of 1,800 mg per day divided into three doses for 4 days followed by 1,200 mg per day for 3 days. Moderate-quality evidence from small randomized trials demonstrates reduction of pain at 48–96 h compared with placebo (Level II randomized trials with methodological limitations) (8).
4. Stool softeners: Fiber psyllium at 3–6 g per day or osmotic laxatives polyethylene glycol at 17 g per day is prescribed.

Expected evolution: Pain peaks within the first 48–72 h followed by gradual decrease. After 2 weeks, most patients are asymptomatic or experience only mild discomfort. The thrombus organizes and reabsorbs, leaving a residual skin tag that is not painful (6).

##### 3.8.1.3 Surgical management: indications within 72 h and severe pain

Surgical treatment is considered first-line in cases presenting within 72 h of onset and accompanied by severe pain. Specific indications include the following:

- duration less than 72 h;
- severe incapacitating pain despite oral analgesia;
- large thrombosis greater than 2 cm or multiple lesions; and
- patient preference after informed consent.

Comparative evidence: A retrospective study (*n* = 340) compared recurrence rates of 3.5% at 12 months following complete elliptical excision with recurrence rates of 12.6% at 12 months after simple incision and clot evacuation (Level III retrospective series) (9). Other studies confirm the superiority of excision over incision, showing lower recurrence rates and fewer symptomatic residual skin tags (6, 9, 11).

##### 3.8.1.4 Surgical technique: elliptical excision (outpatient)

Preparation:

- Anesthesia: local infiltration with lidocaine 1–2% with epinephrine 1:100,000 (volume 3–5 mL), infiltrated in a fan-shaped pattern at the base of the nodule, avoiding injection directly into the thrombus.
- Position: lateral decubitus (Sims) or prone jackknife.
- Antisepsis: preparation with chlorhexidine or povidone iodine.

##### 3.8.1.5 Technical steps

1. Elliptical incision: Using a number 15 scalpel, a fusiform elliptical incision encompassing the skin overlying the thrombus is performed, with 2–3-mm margins orientated radially, parallel to the sphincter. Circumferential incisions are avoided due to risk of stenosis if multiple excisions are required (11).
2. Dissection: Identify and resect the entire thrombosed hemorrhoidal bundle, including the fibrous capsule. Perform blunt dissection with curved scissors until healthy tissue is reached. No organized thrombus remnants should remain as they represent a source of recurrence (9).
3. Hemostasis: Compression with gauze for 2–3 minutes is usually sufficient. Persistent bleeding may require bipolar electrocoagulation or absorbable 4-0 interrupted sutures. Primary closure should be avoided due to the risk of abscess from dead space (11).
4. Open wound healing: Allow heading by secondary intention. Apply petrolatum dressing or topical antibiotic ointment.

Postoperative care:

- NSAIDs for 5–7 days;
- sitz baths three to four times per day;
- stool softeners for 2 weeks;
- return to work in 3–7 days depending on occupation; and
- complete healing in 3–4 weeks.

Complications:

- delayed postoperative bleeding 24–72 h less than 2% (9, 11);
- wound infection less than 1%;
- anal stenosis (exceptional if radial incision is performed); and
- fecal incontinence not reported.

#### 3.8.2 SIHs—Grade IV

Traditionally, acute-phase surgery was avoided due to concerns about stenosis and sepsis. However, a recent meta-analysis (seven studies, *n* = 827) demonstrated that emergency hemorrhoidectomy is safe and reduces recovery time compared with delayed surgery (Level II) (11).

##### 3.8.2.1 Stepwise strategy

1. Manual reduction with sedation and analgesia  

Manual reduction with sedation and analgesia is considered first-line if the duration of symptoms is less than 72 h and necrosis is absent. The objective is to relieve vascular compromise and allow conservative management, postponing elective surgery for 6–8 weeks (8).

##### 3.8.2.2 Technique

- Conscious sedation: Midazolam 2–5 mg IV plus fentanyl 50–100 µg IV is administered in the emergency department.
- Application of osmotic agent: Apply granulated sugar covering the entire prolapsed mass or hypertonic saline solution 20% with soaked gauze for 15–20 min (mechanism: osmotic gradient, extracts interstitial fluid, reducing volume by 30%–50%) (8).
- Gentle manual reduction: Perform circumferential sustained compression with both hands toward the anal canal for 2–5 min. Avoid excessive force due to risk of mucosal tear.
- Outcome: If successful, the mucosa returns to the anal canal and the patient experiences immediate pain relief.

##### 3.8.2.3 After successful reduction

- Hospital observation 24–48 h due to risk of re-prolapse 10%–15%.
- Systemic analgesia plus purified micronized flavonoid fraction (diosmin 1,800 mg per day for 4 days followed by 1,200 mg per day for 3 days according to ESCP 3–2–1 regimen) (8).
- Aggressive stool softening.
- Discharge with elective surgery at 6–8 weeks if stabilization occurs.

Failure of manual reduction — defined as immediate re-prolapse or a persistent irreducible mass after two attempts with adequate sedation—indicates urgent surgery.

##### 3.8.2.4 Emergency hemorrhoidectomy—absolute indications

- Established mucosal necrosis: black-gray areas, deep ulceration, foul odor; risk of sepsis if not debrided (10).
- Uncontrolled bleeding: from mucosal ulceration.
- Failure of manual reduction: despite adequate sedation.
- Incapacitating pain: refractory to systemic analgesia and reduction maneuvers.
- Immunocompromised patient: diabetes, HIV, corticosteroids, or chemotherapy with perianal cellulitis; risk of progression to Fournier gangrene (7).

##### 3.8.2.5 Surgical technique: modified Milligan–Morgan hemorrhoidectomy

The open technique remains the gold standard in emergencies, compared with closed techniques that carry higher risk of abscess formation in edematous necrotic tissue (3, 10).

##### 3.8.2.6 Critical technical principles

- Anesthesia: Regional (spinal or epidural) or general is recommended. Local anesthesia should be avoided because inflamed tissue makes infiltration ineffective.
- Position: Prone jackknife and lithotomy are the best options. Lithotomy is often preferred in emergencies for faster access.
- Identification of bundles: The three main hemorrhoidal bundles are located at positions 3, 7, and 11 o'clock in lithotomy. In SIH grade IV, the edematous mass may obscure anatomy. Careful palpation with forceps helps identify firm vascular pedicles beneath the mucosa.
- Perform a “V”-shaped radial incision in the anoderm, extending from the anal margin to above the dentate line, encompassing the hemorrhoidal bundle. Dissect the vascular pedicle using electrocautery or scissors. Ligate the pedicle with an absorbable 2-0 or 3-0 suture (Vicryl), placing a transfixion stitch at the base to prevent bleeding (3).
- Preservation of mucocutaneous bridges: Maintain at least 1 cm of intact mucosa anoderm between each resection to prevent anal stenosis. If bundles are very close, prioritize resection of the largest and postpone smaller ones (10).
- Meticulous hemostasis using bipolar coagulation of bleeding vessels; perform final inspection with anoscopy or rigid rectoscope.
- Open wounds: no primary suturing; allow healing by secondary intention over four to six weeks.

Technical variant: The use of ultrasonic scalpel or advanced bipolar energy devices may reduce postoperative pain, intraoperative bleeding, and operative time (Level II: small randomized trials in elective hemorrhoidectomy); however, specific data in emergency hemorrhoidectomy settings remain limited (3).

Postoperative care—hospitalization 24–48 h:

- multimodal analgesia including NSAIDs plus paracetamol, with optional weak opioids during the first 48 h;
- sitz baths four to six times daily;
- osmotic laxatives such as polyethylene glycol to maintain soft, formed stool;
- Metronidazole 500 mg every 8 h for 5–7 days; controversial, but some authors use it to reduce odor and theoretical infection risk (10); and
- discharge only when pain is controlled with oral analgesia and first postoperative defecation occurs without complications.

Specific complications in acute phase surgery:

- Postoperative bleeding: first 24 h 5%–8%; most resolve with packing, less than 2% require surgical revision (10).
- Acute urinary retention: 5%–15%, due to pain, sphincter spasm, or spinal anesthesia; transient bladder catheterization may be required.
- Anal stenosis: 2%–5% if inadequate technique (excessive anoderm resection, insufficient bridges) (10).
- Wound infection abscess: 2%–3%.
- Fecal incontinence: less than 1%.

Meta-analysis results: emergency vs. delayed surgery:

- Recovery time: 21 days with emergency surgery vs. 35 days with conservative treatment followed by delayed surgery—a difference of 2 weeks favoring emergency surgery.
- Complications: no significant difference; stenosis 2%–5% in both groups, bleeding 5%–8% in both groups.
- Recurrence at 12 months: no difference, 3%–5% in both groups.
- Level II: meta-analysis of seven observational studies, moderate heterogeneity, with *I*^2^ 45% (10).

### 3.9 Emergency management

Sudden onset prolapse: In a patient without prior hemorrhoidal history, consider intraluminal rectal mass (adenocarcinoma, large polyp, or submucosal tumor) as the origin. Urgent rectosigmoidoscopy should be performed before any reduction or surgery (7).

Fever greater than 38°C, combined with perianal cellulitis and disproportionate pain, raises suspicion of Fournier gangrene or deep abscess and requires urgent CT scan, broad-spectrum antibiotics (piperacillin-tazobactam plus clindamycin), and immediate surgical debridement; mortality is 20–40% if treatment is delayed more than 24 hours (7).

Profuse rectal bleeding: In the context of SIHs, this may indicate arterial ulceration of the hemorrhoidal pedicle. If hemodynamic instability occurs (hypotension, tachycardia), resuscitation with crystalloids transfusion is required, followed by urgent hemorrhoidectomy for bleeding control. Angiography should not delay surgery in unstable patients (3).

### 3.10 Critical surgical points

Surgical timing in THE: The 72-h window is not an absolute rule but a probabilistic guideline. During the first 72 h, most patients remain in the ascending phase of pain, and surgery offers faster relief. After 72 h, most patients experience spontaneous improvement, and surgery does not accelerate recovery. Evaluation must be individualized based on pain intensity, thrombus size, and patient preference (6, 9).

Excision vs. incision in THE: Simple incision with clot evacuation is technically easier and faster (approximately 5 min), but recurrence is 3.5 times higher (12.6% vs. 3.5%) and symptomatic residual skin tags are more frequent if surgery has been performed. Complete elliptical excision of the thrombosed hemorrhoidal bundle should be performed rather than simple drainage (9, 11).

SIH grade IV: The edematous mass may tempt the surgeon to excise all prolapsed tissue in one stage, but this must be avoided. Only the three main bundles should be resected, preserving at least 1 cm of mucosa between resections. Postoperative anal stenosis (2%–5%) is almost always due to excessive anoderm resection where anatomical limits are distorted by edema (10).

Manual reduction in SIH: The use of osmotic agents (granulated sugar or hypertonic solution) is not merely anecdotal but has solid pathophysiological rationale and is recommended by ESCP and ASCRS guidelines. The key is adequate contact time (15–20 min) and appropriate patient sedation, since pain prevents sphincter relaxation, making reduction difficult. If reduction fails after two attempts with adequate sedation, surgery should be performed (8).

Fever: Fever greater than 38°C with perianal cellulitis and disproportionate pain raises suspicion of Fournier gangrene or deep abscess.

Energy devices: Energy devices may reduce pain and bleeding. However, evidence in emergency hemorrhoidectomy remains limited, as most studies involve elective surgery. If the surgeon is experienced, these devices are safe in emergencies. Otherwise, conventional monopolar electrocautery with pedicle ligation remains equally effective (3).

Pregnancy: External hemorrhoidal thrombosis is particularly frequent during the third trimester and immediate postpartum period due to sustained increase in intraabdominal pressure. Conservative management is preferred in the majority of cases. Paracetamol 1 g every 8 h is the first-line analgesic, as NSAIDs are contraindicated in the third trimester due to risk of premature closure of the ductus arteriosus. Purified micronized flavonoid fraction should be used with caution; available data suggest acceptable safety in the second and third trimesters but evidence in the first trimester remains limited. Surgical excision under local anesthesia is not contraindicated in severe cases with incapacitating pain refractory to conservative measures, but requires coordination with obstetrics and anesthesiology. In the immediate postpartum period, spontaneous resolution is frequent and conservative management should be prioritized before considering surgery (12).

## 4 Rectal prolapse—complete and mucosal

### 4.1 Definition and pathophysiology

Complete rectal prolapse: This involves intussusception of the full thickness of the rectum that protrudes through the anal canal with exteriorization of all layers of the rectal wall (13). Anatomically, it results from the combination of failure of rectosacral fixation, diastasis of the levator ani muscle, deep pouch of Douglas, and weakness of the sphincter complex (13, 14).

Mucosal rectal prolapse: This involves partial protrusion of mucosa with or without submucosa, without full-thickness exteriorization of the rectal wall. It is associated with mucosal laxity, chronic straining, and frequently coexists with hemorrhoidal disease and mild sphincter incompetence (15). The predominant mechanism is sliding of the mucosa and hemorrhoidal cushion, without the full-thickness intussusception pattern typical of complete prolapse (15).

### 4.2 Epidemiology and risk factors

Complete rectal prolapse: The estimated prevalence is 0.5% in the general population, with female predominance (ratio 6:1) and peak incidence in the seventh decade of life (13, 14). Risk factors include advanced age, multiparity, chronic constipation with excessive straining, pudendal denervation, and connective tissue disorders (13, 14).

Mucosal prolapse: This is predominantly observed in the context of defecatory disorders (chronic straining, constipation, or diarrhea), with significant clinical overlap with advanced hemorrhoids (14, 15). Its prevalence as an isolated entity is difficult to estimate due to heterogeneous classification in surgical series.

### 4.3 Clinical manifestations

Complete rectal prolapse: The classic triad includes circumferential protruding mass during or after defecation, mucous discharge with perianal maceration, and mild-to-moderate rectal bleeding. Fecal incontinence coexists in 41%–83% of cases, depending on the series and surgical technique, secondary to chronic dilation of the anal canal and pudendal denervation (16, 17). Paradoxical constipation occurs in 30%–50% of cases due to evacuation obstruction associated with intussusception (17).

Current controversy: Debate persists on whether complete prolapse causes or results from fecal incontinence. Manometric studies suggest pudendal denervation may precede prolapse in some patients. This may explain incomplete continence recovery after repair: improvement in 60%–70% vs. complete resolution in 30%–40% (16, 18).

Mucosal prolapse shares symptoms with complete prolapse (bleeding, mucus, pruritus, and sensation of a mass) but is less bulky and usually associated with prolapsed hemorrhoids (15). True fecal incontinence is rare (less than 10%), while mucous soiling predominates. Functional severity is lower than in complete prolapse, although pelvic floor dysfunction may coexist (15, 18).

### 4.4 Diagnosis

Complete rectal prolapse: Clinical diagnosis is made by inspection during the Valsalva maneuver or in the squatting position. The pathognomonic finding is circumferential concentric mucosal folds with absence of a sulcus between the prolapsed rectal wall and the anal skin, which differentiates complete prolapse from mucosal and hemorrhoidal prolapse (13).

Mucosal prolapse: It is characterized by radial folds rather than concentric folds, with eversion of the anoderm and a sulcus between the rectum and anal skin. Overlap with prolapsed internal hemorrhoids is common. When clinical doubt exists, endoscopy with biopsy is mandatory to exclude neoplastic lesions (13–15).

Complementary studies are of critical importance, with specific indications for each of them as stated in Table 2.

**Table 2:** Complementary studies and indications in complete rectal prolapse vs. mucosal prolapse.

Study	Complete rectal prolapse indication	Mucosal rectal prolapse indication
Dynamic defecography (MRI/fluoroscopy)	Occult prolapse, enterocele, rectocele, complex surgical planning	Only if symptoms of obstructed defecation or pelvic floor dysfunction
Anorectal manometry	Evaluates baseline sphincter function; greater prognostic impact before surgery	Useful if incontinence or soiling, to differentiate sphincter dysfunction
Colonoscopy	Sudden onset, significant bleeding, suspected intraluminal mass	Always if atypical, ulcerated mucosal lesion, or suspicion of neoplasia

MRI, magnetic resonance imaging.

### 4.5 Treatment

General principle: Complete rectal prolapse requires surgical repair in symptomatic patients who are fit for surgery. Conservative management does not correct the anatomical defect, although it may palliate symptoms (Level I: multiple randomized controlled trials and systematic reviews) (13, 19). Mucosal prolapse usually responds to less invasive strategies, reserving surgery for persistent or circumferential cases (15).

#### 4.5.1 Abdominal approach—complete prolapse

Indications: surgically fit patients (American Society of Anesthesiologists [ASA] score ≤3, age <75–80 years, life expectancy >5 years) (17, 19).

- Techniques:

Rectopexy with mesh, also known as the Wells technique, involves anterior fixation to the sacral promontory, preserving posterior dissection. Reported recurrence rates are between 4% and 8% at 5 years. *De novo* constipation occurs in 15% to 20% of patients, which is lower than posterior rectopexy. Continence improves in 60%–70% of cases (Level II: prospective multicenter series) (17, 20). The modified Wells procedure (Ivalon sponge rectopexy) involves posterior fixation of the rectum to the presacral fascia using a polyvinyl alcohol (Ivalon) sponge wrapped around the posterior and lateral walls of the rectum. While historically significant and associated with recurrence rates of 2%–10%, its use has declined due to concerns over sponge infection and erosion. The Ripstein procedure involves circumferential or anterior mesh fixation of the anterior rectal wall to the sacral promontory. It provides secure fixation but carries a risk of obstructive defecation (10%–20%) due to anterior mesh compression, and has largely been replaced by ventral rectopexy in contemporary practice (19, 20). In patients with documented preoperative constipation or symptomatic dolichomegacolon, concomitant sigmoid colon resection (resection rectopexy) should be explicitly considered and discussed, as it significantly reduces postoperative constipation rates compared with rectopexy alone (15% vs. 30%–40%). This combined approach is particularly indicated when preoperative colonic transit studies confirm slow-transit constipation or when dolichomegacolon is identified on imaging (17, 20).

- Laparoscopic ventral rectopexy with mesh (D'Hoore technique):

This involves an anterior-only dissection of the rectovaginal or rectoprostatic space with anterior mesh fixation to the sacral promontory, avoiding posterior dissection and preserving autonomic innervation. Currently, it is the most widely performed abdominal technique for complete rectal prolapse in surgically fit patients. It has a recurrence rate of 4%–8% at 5 years. *De novo* constipation occurs in 8%–12% of patients, significantly lower than posterior rectopexy due to preservation of posterior innervation. Continence improves in 60%–70% of patients (Level I–II: systematic review and meta-analysis) (20).

- Ventral rectopexy without mesh (Orr–Loygue technique):

This is an alternative technique when prosthetic material must be avoided (prior mesh infection, young patients, or surgeon preference). It has a recurrence rate of 10%–15%, with a lower risk of mesh erosion or infection (Level II) (20).

Comparative results (meta-analysis): Abdominal rectopexy has a recurrence rate of 6% (95% CI 4%–9%), whereas perineal approaches have a recurrence rate of 19% (95% CI 14%–25%). Abdominal perioperative procedures are associated with a mortality of 0.5%–2% (Level I: meta-analysis) (13, 19).

In cases of mucosal prolapse, abdominal rectopexy is rarely considered first-line treatment. The objective is treatment of the mucosal or hemorrhoidal component through local or perineal procedures (15).

#### 4.5.2 Perineal approach

For complete prolapse (frail patient or short prolapse):

- Altemeier procedure (perineal proctosigmoidectomy): This is indicated in frail patients (ASA ≥4) or in emergency settings. Reported perioperative mortality rates range from 0% to 2%, recurrence occurs in 15%–30% of patients, and reoperation carries low risk. Continence improves in 40%–60% of patients (Level II) (19, 21).
- Delorme procedure (mucosectomy with plication): This is reserved for short prolapses (<5 cm) and patients with high surgical risk (ASA ≥3 with significant comorbidities or advanced age) who are not candidates for mesh implantation (21).

For persistent or circumferential mucosal prolapse:

- First–line treatment: fiber supplementation, stool softeners, and defecatory retraining for at least 8–12 weeks (Level V: expert opinion) (15).
- Ambulatory procedures: rubber band ligation, sclerotherapy, or radiofrequency ablation considered when a hemorrhoidal component coexists (Level II–III: prospective series) (15).
- Refractory circumferential mucosal prolapse: Delorme mucosectomy as a low morbidity perineal option, with risk of anal stenosis with PPH (procedure for prolapse and hemorrhoids) 3–8%; reserve when the hemorrhoidal component predominates (15).

### 4.6 Emergency management

Irreducible complete prolapse requires urgent reduction with evaluation of tissue viability, along with the following (22):

- adequate sedation and analgesia;
- osmotic reduction of edema with application of granulated sugar over the prolapsed mucosa for 15–30 min (Level IV: case series) (22);
- gentle sustained circumferential manual compression;
- if reduction successful, elective surgery deferred for 24–48 h under better conditions; and
- if reduction fails or signs of ischemia or necrosis are present, urgent surgery, generally using the Altemeier procedure (Level V: expert opinion) (22).

### 4.7 Critical surgical points

- Urgent surgery: This is necessary when complete prolapse is irreducible for more than 6 h with ischemic changes (pallor, cyanosis, or necrosis). It requires reduction with osmotic granulated sugar. If reduction fails or necrosis is established, surgery must not be delayed. Altemeier is the preferred approach in emergency settings. Mucosal prolapse rarely produces this emergency; if suspected, misclassified complete prolapse should be excluded (22).
- Approach selection in complete prolapse: In surgically fit patients (ASA ≤3, age <75 years), laparoscopic or robotic ventral rectopexy is preferred, offering a recurrence rate of 6% compared with 19% for perineal approaches, and continence improvement in 60%−70% of patients. In high-risk patients (ASA ≥4, age >75−80 years), the Altemeier or Delorme procedure is recommended, accepting higher recurrence rates but with the possibility of low-risk reintervention if needed; perioperative mortality ranges from 0%−2% for perineal vs. 0.5%−2% for abdominal approaches (19, 21).
- Prevention of *de novo* constipation: In such circumstances, ventral rectopexy is preferred rather than posterior. Extensive posterior dissection denudes autonomic innervation and increases constipation risk from 15% to 30%. In patients with severe baseline constipation, concomitant sigmoidectomy must be considered when symptomatic dolichosigma is present (17, 20). When dolichomegacolon is identified preoperatively (whether on imaging or colonic transit studies), sigmoid colon resection should be performed concomitantly with rectopexy (resection rectopexy, Frykman–Goldberg procedure). This combined approach reduces postoperative constipation rates and is strongly recommended in patients with preoperative constipation, as rectopexy alone may worsen or perpetuate constipation by straightening and kinking the sigmoid loop without removing the redundant segment.
- Intraoperative differentiation mucosal vs. complete prolapse: If protruding mucosa appears polypoid, ulcerated, or resistant to manual reduction, biopsy should be obtained before completing resection. Complete prolapse includes all palpable layers, whereas mucosal prolapse yields to gentle digital pressure without an external muscular component (13, 14).
- Management of persistent incontinence after repair: If no improvement is observed at 6 months, anorectal manometry should be performed to evaluate residual sphincter function, and anatomical recurrence through dynamic proctography should be excluded. Sacral neuromodulation or secondary sphincteroplasty can be considered only after confirming the absence of recurrence (17, 18).
- Therapeutic escalation in mucosal prolapse: Surgery should never be performed without at least 8–12 weeks of conservative measures. In refractory circumferential mucosal prolapse, the perineal Delorme procedure offers lower morbidity than circular hemorrhoidectomy (PPH), with anal stenosis risk of 3%–8% (15).

## 5 Anal condylomas

### 5.1 Definition and pathophysiology

Anal condyloma acuminatum are benign epithelial proliferations caused by infection with low-oncogenic-risk human papillomavirus (HPV), predominantly types 6 and 11 (accounting for more than 90% of cases) (23, 24). Histologically, they correspond to epithelial hyperplasia with koilocytosis (cells with perinuclear halos and irregular nuclei) (23).

### 5.2 Epidemiology and risk factors

Global prevalence ranges from 0.5% to 33%, depending on the population studied, with the highest burden among men who have sex with men and individuals living with HIV (25, 26). The annual incidence among HIV-positive men who have sex with men reaches 15%–20%, with recurrence after treatment ranging from 30% to 70%, depending on the modality used (26, 27).

Risk factors include receptive anal intercourse, multiple sexual partners, coexistence of other sexually transmitted infections, immunosuppression (HIV, transplantation, or immunosuppressive therapy), and smoking (24, 26). The presence of external perianal warts is associated with intraanal involvement in 60%–80% of cases (28).

### 5.3 Clinical manifestations

Anal condylomas present as exophytic papules measuring 1–10 mm, skin-colored or erythematous, with a verrucous surface, which may coalesce to form cauliflower-like plaques (23). Symptoms include pruritus (60%), mucous discharge (40%), mild postdefecatory bleeding (30%), and foreign body sensation (23, 24).

In patients with HIV, particularly those with CD4 counts below 200 cells/mm^3^, lesions tend to be more extensive (greater than 10 cm^2^), confluent, and refractory to treatment (26, 27).

Current controversy: HPV typing is not recommended for management of clinically evident condylomas because it does not modify therapeutic decisions (24). However, debate persists regarding screening for high-grade anal intraepithelial neoplasia in high-risk populations (HIV-positive individuals and those with history of cervical or vulvar dysplasia) using anal cytology and high-resolution anoscopy (29). Current guidelines recommend high-resolution anoscopy in selected populations while acknowledging limitations in resources and training (Level II: cohort studies) (29).

### 5.4 Diagnosis

Diagnosis of anal condylomas is clinical in more than 95% of cases. Biopsy is reserved for atypical lesions, such as those that are ulcerated, indurated, pigmented, fixed to deep planes, fail to respond after 3 months of standard therapy, show rapid growth, or occur in immunosuppressed patients with extensive lesions (23, 24, 29).

Systematic anoscopy is indicated in all patients with perianal condylomas to evaluate intraanal extension (28). High-resolution anoscopy is reserved for high-oncogenic-risk populations, depending on availability (29).

The differential diagnosis of perianal condylomas includes anal skin tags (fibroepithelial polyps of the perianal skin), which are soft, painless, non-verrucous, pedunculated lesions without viral etiology and do not require treatment unless symptomatic. Perianal malignancies, particularly squamous cell carcinoma, extramammary Paget's disease, and anal melanoma, must be excluded by biopsy in any lesion presenting with ulceration, induration, pigmentation, fixation to deep planes, or rapid growth (23, 29).

### 5.5 Treatment

No treatment guarantees viral eradication; the goal is clearance of lesions, accepting recurrence as part of the natural course of the disease (23, 30).

#### 5.5.1 Self-administered treatments suitable only for small external lesions less than 10 cm^2^

1. Imiquimod 5% cream: application three times per week for 16 weeks; complete clearance in 35%–50% cases (Level I: randomized controlled trials) (23, 24).
2. Podophyllotoxin 0.5% gel: application twice daily for 3 days, followed by 4 days of rest, up to 4 cycles; clearance in 45%–80% of cases(Level I) (24).

Contraindications include intraanal lesions, pregnancy, and areas greater than 10 cm^2^

#### 5.5.2 Provider-administered treatments: intraanal, extensive, or refractory lesions

1. Simple surgical excision: resection with scissors or scalpel under local or regional anesthesia; initial clearance in 70%–90% of cases, recurrence in 20%–30% of cases at 1 year (Level II: prospective series) (23, 30).
2. Electrosurgery (electrocautery, electrofulguration, or thermal destruction): similar results to excision; higher risk of anal stenosis if more than 50% of the circumference is treated (Level II) (23, 30).
3. CO₂ laser selective vaporization: advantages include less bleeding and precision in intraanal lesions; disadvantages include cost, availability, and risk of viral aerosol transmission requiring smoke evacuation; clearance achieved in 60%–90% of cases, with recurrence in 20%–40% of patients (Level II) (30).

Combined therapy with excision plus adjuvant imiquimod reduces recurrence compared with excision alone (18% vs. 35%, *p* = 0.02) in limited series (Level III: retrospective studies) (30).

Follow-up: Follow-up should include evaluation every 3 months during the first year after treatment for early detection of recurrence (24).

### 5.6 Emergency management

Not applicable: Anal condylomas do not generate surgical emergencies by themselves. However, extensive lesions with secondary bacterial infection (perianal cellulitis) require systemic antibiotics and drainage if a purulent collection is present.

### 5.7 Critical surgical points

Mandatory biopsy is required for lesions with ulceration, induration, fixation to deep planes, or atypical pigmentation. In such cases, complete excisional biopsy should be performed rather than superficial punch biopsy in order to exclude invasive squamous cell carcinoma. The specimen should be oriented with silk suture for margin evaluation; superficial biopsy may be falsely negative if carcinoma is located at the base of the lesion.

To prevent anal stenosis, no more than 50% of the anal circumference should be treated in a single procedure using electrocautery or laser. If lesions involve more than 180°, treatment should be staged in two or three sessions separated by 4–6 weeks to allow re-epithelialization. Postoperative stenosis should be monitored beginning 1 month after treatment.

Screening is essential in high-risk populations. HIV-positive patients with CD4 counts below 200 cells/mm^3^ or those with a history of high-grade cervical or vulvar dysplasia require annual high-resolution anoscopy for detection of high-grade anal intraepithelial neoplasia, regardless of the presence of visible condylomas. Progression of AIN3 to invasive carcinoma occurs in approximately 10% of HIV-positive individuals within 5 years.

Management of extensive intraanal lesions requires special consideration. When condylomas extend more than 3 cm proximal to the dentate line, treatment should be performed under regional or general anesthesia with anal retractors such as Lone Star or Parks to ensure adequate exposure. Excision must include the entire implantation base; superficial fulguration without excision has recurrence rates greater than 60% in intraanal lesions.

## 6 Hypertrophied anal Papilla

### 6.1 Definition and pathophysiology

Hypertrophied anal papilla corresponds to reactive hypertrophy of an anatomical papilla at the dentate line, secondary to chronic mechanical or inflammatory stimulation (31, 32). Histopathologically, it represents a fibroepithelial polyp with a vascularized connective tissue core covered by stratified squamous or transitional columnar epithelium (33).

### 6.2 Epidemiology and risk factors

Hypertrophied anal papilla is associated with chronic anal fissure in more than 90% of cases, forming part of the chronicity triad together with induration of the fissure edges and a distal sentinel skin tag (34, 35). Other contributing factors include chronic constipation, chronic diarrhea, and repeated defecatory microtrauma (32, 35).

### 6.3 Clinical manifestations

Most lesions are asymptomatic and discovered incidentally during anoscopy. When they reach sizes greater than 1–2 cm, they may produce a prolapse sensation during or after defecation, foreign body sensation, pruritus due to difficulty with perianal hygiene, and mild bleeding secondary to trauma (31, 32).

The clinical differential diagnosis includes prolapsed internal hemorrhoids, anal canal polyps, and less frequently neoplastic lesions. In referral series to coloproctology, up to 6% of lesions initially classified as hemorrhoids corresponded to hypertrophied anal papilla (36).

### 6.4 Diagnosis

Diagnosis is clinical, established by anoscopy. The typical finding is a single pedunculated lesion with its base at the dentate line, generally posterior at the 6 o'clock position in lithotomy, corresponding to the typical location of anal fissures. The lesion has a firm elastic texture and is mobile on its pedicle (31, 32). Association with chronic anal fissure is diagnostic in more than 90% of cases (34, 35). Atypical lesions (multiple, sessile, ulcerated, indurated, or larger than 3 cm) require biopsy to exclude cloacogenic polyp or anal intraepithelial neoplasia (37, 38).

### 6.5 Treatment

Asymptomatic hypertrophied anal papilla associated with acute fissure: This is managed conservatively, focusing on treatment with stool softeners and topical analgesia. The papilla may regress once the inflammation resolves (Level V expert opinion) (35).

Asymptomatic hypertrophied anal papilla associated with chronic fissure: In such cases, observation is acceptable, or excision can be performed during lateral sphincterotomy, depending on surgeon and patient preference. Concomitant resection improves cosmetic satisfaction without increasing complications (Level III retrospective series) (31, 34, 39).

Symptomatic papillae (any context): These require transanal excision under local or regional anesthesia. The technique involves traction of the papilla, ligation of the base with absorbable 3-0 suture, resection with scissors or scalpel, and hemostasis. Reported complications include bleeding in less than 2%, infection in less than 1%, and recurrence in less than 5% (Level III) (31, 34).

Current controversy: There is no consensus regarding routine vs. selective excision of hypertrophied anal papilla during lateral sphincterotomy for chronic fissure. Retrospective studies suggest that resection improves patient satisfaction without increasing complications, but randomized controlled trials are lacking (31, 34, 39).

### 6.6 Emergency management

Not applicable: Hypertrophied anal papilla does not generate surgical emergencies. However, significant bleeding due to trauma of a large papilla may require urgent hemostatic ligation under local anesthesia.

### 6.7 Critical surgical points

- Timing of resection during lateral sphincterotomy for chronic fissure: Concomitant excision of hypertrophied anal papilla improves cosmetic satisfaction without increasing complications of bleeding, infection, or transient incontinence. The pedicle base should be ligated with absorbable suture (3-0 or 2-0 Vicryl or chromic) before resection to prevent bleeding from the vascular axis, and meticulous hemostasis of the bed is required. Delayed bleeding (within 12–24 h) is the most frequent complication.
- Intraoperative differential diagnosis: If the papilla is sessile, and not pedunculated, during examination under anesthesia, has an implantation base larger than 1.5 cm, is fixed to deep planes, or measures larger than 3 cm, frozen section biopsy should be considered to exclude cloacogenic polyp or anal intraepithelial neoplasia before completing radical resection. Typical hypertrophied anal papilla is always pedunculated and mobile, whereas sessile lesions suggest alternative pathology.
- Management of multiple papillae: The presence of two or more hypertrophied papillae without association with anal fissure is atypical and requires exclusion of inflammatory bowel disease (Crohn's), chronic proctitis, and synchronous neoplastic process. Complete anoscopy and rectosigmoidoscopy should be performed, and all lesions must be sent for histopathology before discharge.
- Prevention of recurrence: Recurrence of hypertrophied anal papilla after excision is less than 5% when the underlying anal fissure is adequately treated with lateral sphincterotomy or botulinum toxin. Isolated papilla resection without treatment of the fissure has a recurrence rate greater than 40%, as the chronic inflammatory stimulus persists.

## 7 Anal canal polyps

### 7.1 Definition and pathophysiology

Anal canal polyps include four histological entities: fibroepithelial polyps, hypertrophied papillae with greater fibrous component, inflammatory cloacogenic polyps, and anal canal adenomas (32, 33).

### 7.2 Epidemiology and risk factors

No clearly defined specific risk factors exist, although anal canal adenomas share risk factors with colorectal neoplasia, including age over 50 years, family history of colorectal cancer, and Lynch syndrome.

### 7.3 Clinical manifestations

Clinically, these lesions may be pedunculated or sessile, single or multiple, and often protrude during defecation. Symptoms include intermittent prolapse, mucous discharge, mild bleeding, and a sensation of incomplete evacuation (33).

### 7.4 Diagnosis

Diagnosis is made by anoscopy, which identifies the location (generally 0–2 cm above the dentate line) and macroscopic characteristics. Colonoscopy is required to exclude proximal synchronous lesions, especially in adenomatous polyps (40).

Biopsy or polypectomy is mandatory for histological classification and exclusion of dysplasia or malignancy (40).

Low rectal polyps originating 2–10 cm above the dentate line may prolapse through the anal orifice and clinically mimic anal canal polyps, particularly when pedunculated and large. Distinguishing features include a longer pedicle, greater size, and origin above the level detectable by anoscopy alone. Colonoscopy is mandatory in any patient presenting with a prolapsing polyp that cannot be fully visualized or attributed to the anal canal by anoscopy, as low rectal polyps carry a higher risk of advanced dysplasia and malignant transformation than true anal canal polyps and require endoscopic or surgical resection with adequate margins and subsequent surveillance (40).

### 7.5 Treatment

Treatment depends on the different types of polyps. Fibroepithelial or inflammatory polyps are managed with endoscopic polypectomy using a snare or transanal excision, with recurrence rates of less than 5% (Level III) (33).

Anal canal adenomas require complete resection with margins, due to their association with rectal adenocarcinoma. Approximately 5–10% contain foci of high-grade dysplasia or intramucosal carcinoma (Level III) (41).

### 7.6 Emergency management

Not applicable: Anal canal polyps do not generate surgical emergencies**.**

### 7.7 Critical surgical points

- Resection with margins in adenomas: Anal canal adenomas carry a 5–10% risk of high-grade dysplasia and 2–3% risk of intramucosal carcinoma.. Resection should be performed with a circumferential margin of 3–5 mm of healthy mucosa. The specimen should be oriented with silk suture for histopathological margin evaluation. If margins are involved on final pathology, re-resection should be performed in 4–6 weeks.
- Intraoperative differentiation: Fibroepithelial polyps are soft, mobile, and smooth. Inflammatory cloacogenic polyps are firm with a granular or eroded surface. Adenomas have a lobulated, friable surface. When in doubt, complete excisional biopsy is required, as forceps biopsy may be insufficient to differentiate adenoma from intramucosal adenocarcinoma.
- Synchronous colonoscopy: Every patient with anal canal adenoma requires complete colonoscopy within 3–6 months to exclude synchronous colonic neoplasia, as up to 30% present synchronous adenomatous polyps in the colon. Endoscopic surveillance should be initiated according to colorectal cancer prevention guidelines.
- High-grade dysplasia on initial biopsy requires wide local excision with 5 mm margins and multidisciplinary oncological evaluation before definitive management.

## 8 Discussion

**Figure 2:**
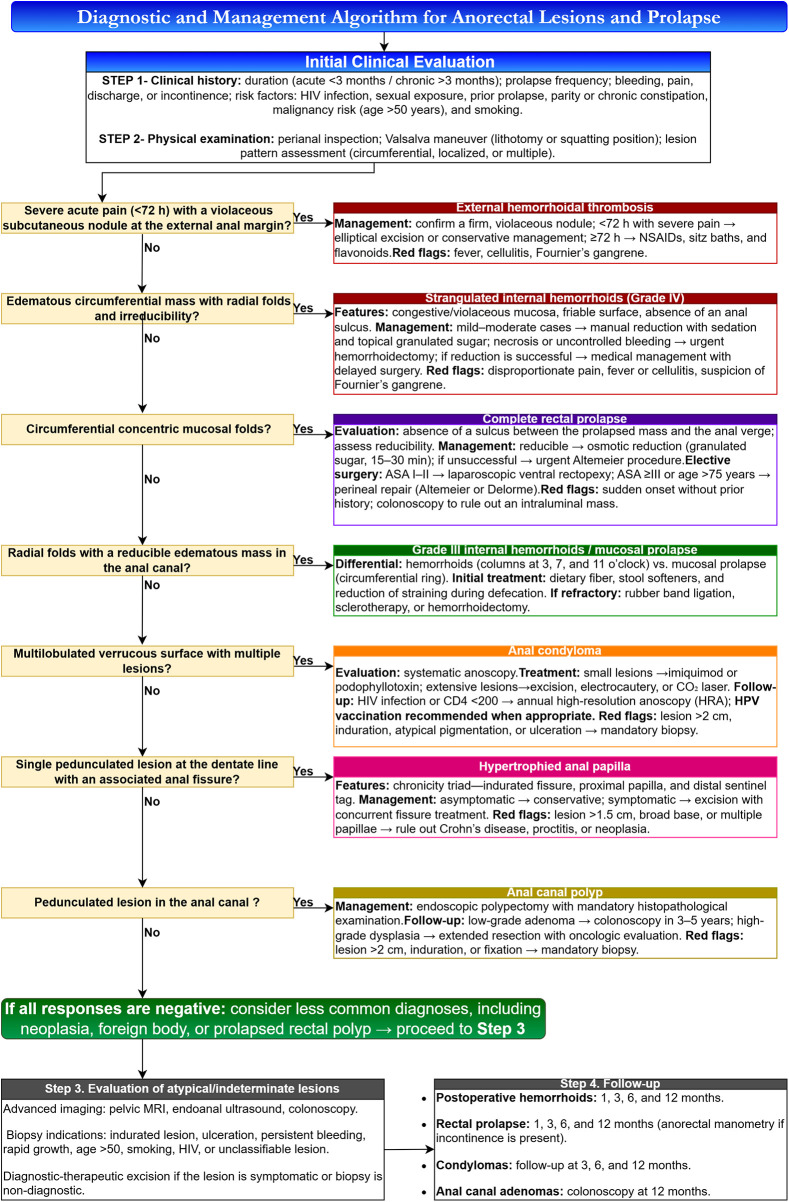
Diagnostic differential and surgical management algorithm for benign prolapsing anal lesions based on physical examination findings.

This review integrates the current evidence on benign prolapsing anal lesions within an operational framework based on objective clinical findings. The proposed algorithm (Figure 2) prioritizes targeted physical examination as the first-line diagnostic tool, relegating imaging studies to the characterization of submucosal lesions or surgical planning, consistent with principles of high-value medicine. The algorithm intentionally uses pain as the initial branching criterion to prioritize the rapid identification of surgical emergencies such as hemorrhoidal thrombosis, strangulated internal hemorrhoids, and ischemic rectal prolapse, which require urgent intervention. Entities such as internal hemorrhoidal prolapse, hypertrophied anal papilla, and anal canal polyps, which are typically painless, are reached through the “pain absent” branch of the algorithm, reflecting their non-urgent clinical presentation. This structure follows the principle of ruling out time-sensitive conditions before proceeding to elective differential diagnosis. It must be explicitly acknowledged that the proposed diagnostic algorithm represents an expert opinion-based construct (Oxford Level V), derived from synthesis of the available literature and clinical experience. It has not been prospectively validated in independent cohorts, and therefore its applicability should be interpreted within this methodological limitation. Table 1 summarizes the key differential findings that allow bedside diagnostic orientation, while Table 2 provides the specific indications for complementary studies in the context of rectal prolapse. Representative clinical images of each entity (Figure 1) illustrate the morphological patterns described in the text and reinforce the value of physical examination as the central diagnostic tool.

### 8.1 Limitations of the current evidence

The methodological quality of the literature in this field presents significant limitations. For acute hemorrhoids, the evidence comparing excision vs. incision in thrombosed external hemorrhoids derives from single-center retrospective series with modest sample sizes (*n* = 50–340) and heterogeneous definitions of recurrence (6, 9, 11). No randomized controlled trials rigorously comparing these techniques currently exist. For incarcerated internal hemorrhoids, the meta-analysis evaluating urgent hemorrhoidectomy included seven observational studies (six retrospective and one prospective non-randomized) with moderate heterogeneity in the definition of urgency and in the surgical techniques used (11). The absence of randomized controlled trials reflects ethical and logistical challenges in randomizing patients presenting with severe pain.

For rectal prolapse, although meta-analyses of surgical techniques are available, most compare retrospective series with heterogeneity in outcome definitions (such as recurrence and continence) and in duration of follow-up (17, 19, 20). Randomized controlled trials are scarce and limited by small sample sizes and substantial loss to follow-up. The lack of consensus regarding patient-centered outcomes such as quality of life and satisfaction vs. surgeon-centered outcomes such as anatomical recurrence complicates evidence synthesis.

For anal condylomas, high-quality evidence is largely limited to self-administered topical treatments such as imiquimod and podophyllotoxin supported by well-designed randomized controlled trials (21, 24). However, direct comparisons between surgical modalities (such as excision, electrocautery, and laser) lack randomized trials and recommendations rely primarily on case series with potential selection bias. The heterogeneity of studied populations—including HIV-positive vs. HIV-negative patients, extent of lesions, and previous treatments—further limits generalizability of results.

Table 3 summarizes the key clinical recommendations derived from this review with their corresponding level of evidence and grade of recommendation according to the Oxford Centre for Evidence-Based Medicine classification (2011). This overview allows rapid identification of recommendations with strong supporting evidence vs. those relying on expert opinion or small retrospective series, which require prospective validation.

**Table 3:** Summary of key recommendations with level of evidence (LoE) and grade of recommendation according to Oxford Centre for Evidence-Based Medicine classification 2011.

Recommendation	Clinical context	LoE (Oxford)	Grade
Elliptical excision superior to incision alone in thrombosed external hemorrhoids <72 h (recurrence: 3.5% vs. 12.6%)	Acute thrombosed external hemorrhoid	III	B
Emergency hemorrhoidectomy reduces recovery time vs. delayed surgery in grade IV strangulated hemorrhoids without necrosis	Strangulated internal hemorrhoids grade IV	II	B
Abdominal rectopexy has lower recurrence than perineal approach (6% vs. 19%) in complete rectal prolapse	Complete rectal prolapse, fit patient	I	A
Altemeier procedure preferred in frail patients (ASA ≥4) or emergency prolapse reduction failure	Complete rectal prolapse, high surgical risk	II	B
Imiquimod 5% and podophyllotoxin 0.5% are effective first-line topical treatments for small external condylomas	Anal condylomas <10 cm^2^	I	A
No more than 50% of anal circumference should be treated with electrocautery or laser in a single session to prevent stenosis	Extensive condylomas requiring ablation	V	D
Symptomatic hypertrophied anal papilla should be excised; asymptomatic papilla may be observed or excised concomitantly with lateral sphincterotomy	Hypertrophied anal papilla	III	C
All anal canal polyps require histological classification; adenomas mandate complete resection with margins and colonoscopic surveillance	Anal canal polyps	III	C
Diagnostic algorithm for prolapsing anal lesions (Figure 2) is expert opinion-based and requires prospective validation	All prolapsing anal lesions	V	D

Grade A, consistent Level I evidence; Grade B, consistent Level II or III evidence; Grade C, Level IV evidence or extrapolation from Level II/III; Grade D, Level V or inconsistent evidence.

### 8.2 Areas of uncertainty and controversies

1. Management of coexistence of pathologies: Patients frequently present simultaneously with rectal prolapse, hemorrhoids, and pelvic floor dysfunction. The optimal treatment sequence—addressing all conditions in a single stage or through staged procedures—lacks comparative evidence.
2. Prevention of recurrence in condylomas: Adjuvant therapy with imiquimod after excision shows promising results but requires validation in adequately designed randomized controlled trials (30).
3. Screening for anal intraepithelial neoplasia in high-risk populations: Although high-resolution anoscopy detects premalignant lesions, effectiveness studies demonstrating reduction in anal cancer incidence or mortality through screening programs similar to those established for cervical cancer are lacking (29).
4. Optimal surgical timing in thrombosed external hemorrhoids: The 72-h rule represents a pragmatic simplification; however, some patients present at approximately 60 h with plateau pain that has not yet declined and benefit from surgery, while others present at around 50 h with pain already improving and do not require intervention. Prospective studies evaluating inflammatory biomarkers or validated pain scales are needed to better predict which patients benefit from surgery independent of time from symptom onset.

### 8.3 Future directions

Multicenter randomized controlled trials are needed to compare the following:

1. Excision vs. incision in thrombosed external hemorrhoids within 72 h with recurrence at 12 months defined using standardized criteria as the primary outcome.
2. Urgent hemorrhoidectomy vs. manual reduction followed by delayed surgery in incarcerated internal hemorrhoids without necrosis, with recovery time to normal activities as the primary outcome.
3. Laparoscopic vs. robotic ventral rectopexy, with recurrence, continence, and quality of life as outcomes.
4. Excision plus imiquimod vs. excision alone in anal condylomas, with recurrence at 24 months as the primary outcome.
5. Routine vs. selective excision of hypertrophied anal papilla during lateral sphincterotomy, with patient satisfaction and complication rates as outcomes.

### 8.4 Comparison with existing guidelines

The recommendations derived from this review are broadly consistent with, but extend beyond, the scope of existing specialty guidelines. The ASCRS clinical practice guidelines for hemorrhoidal disease (3) and the ESCP guideline (8) address hemorrhoidal management comprehensively but do not provide an integrated differential diagnostic algorithm encompassing the full spectrum of prolapsing anal lesions. Similarly, the WSES-AAST (World Society of Emergency Surgery – American Association for the Surgery of Trauma) anorectal emergencies guidelines (22) focus on acute presentations without offering a structured framework for ambulatory differentiation. The 2021 Sexually Transmitted Infections Treatment Guidelines (CDC/MMWR) (23) and the IUSTI-Europe guideline for anogenital warts (23) address condyloma management but are not designed to guide surgical differentiation from other prolapsing entities. The ACG (American College of Gastroenterology) clinical guidelines for benign anorectal disorders (35) provide management recommendations for individual conditions but lack an integrated diagnostic algorithm. The present review addresses this gap by synthesizing guidance from all these sources into a single clinical decision framework, while explicitly acknowledging where recommendations derive from expert consensus rather than high-level evidence. Where our recommendations diverge from or expand upon existing guidelines such as the stepwise osmotic reduction protocol for strangulated hemorrhoids and the staged treatment approach for circumferential condylomas, the underlying evidence base and its limitations are transparently described.

### 8.5 Limitations of this review

Beyond the limitations of the primary evidence described earlier, this review carries several inherent methodological constraints that must be acknowledged. First, the narrative design, while appropriate for the integrative and educational objectives of this work, does not follow the structured methodology of a systematic review. As a result, selection bias in article inclusion cannot be fully excluded despite our explicit selection criteria. Second, several clinical recommendations, particularly those concerning hypertrophied anal papilla and anal canal polyps, rest on small retrospective series or case series (*n* ≥ 10), representing Oxford Level IV evidence at best. Readers should interpret these recommendations with appropriate caution. Third, and most critically, the proposed diagnostic algorithm has not been prospectively validated in an independent clinical cohort. Its diagnostic accuracy, interobserver reliability, and impact on clinical outcomes remain unknown. Prospective validation studies, ideally multicenter, are required before the algorithm can be recommended for broad implementation. Fourth, the restriction to literature in English, Spanish, and Italian may have introduced language bias, potentially excluding relevant studies published in other languages. Fifth, the lack of a formal PRISMA flowchart, while consistent with the narrative design, limits the reproducibility of the search and selection process. These limitations are explicitly outlined to contextualize the level of confidence that should be attributed to the conclusions presented.

Sixth, certain entities that may occasionally mimic prolapsing anal lesions were not included in this review. These include mucosal ectropion secondary to coloanal anastomosis or Whitehead hemorrhoidectomy, and ectropion following imperforate anus repair. The latter predominantly affects pediatric populations outside the scope of this adult-focused narrative, while the former two represent postoperative anatomical sequelae rather than primary prolapsing lesions requiring differential diagnosis in the acute setting.

## 9 Conclusions

Benign prolapsing anal lesions represent a heterogeneous group of conditions whose differential diagnosis relies primarily on physical examination findings, including fold pattern, surface texture, mobility, and anatomical location. The proposed algorithm integrates these findings into operational decision sequences, optimizing the use of complementary studies and guiding therapeutic management according to the available level of evidence.

Systematic identification of red flags allows timely exclusion of malignant pathology or surgical emergencies, minimizing the risk of diagnostic delay. The critical surgical points provide specific technical guidance for surgeons regarding intraoperative decision-making, complication prevention, and optimization of functional outcomes.

However, the quality of evidence supporting therapeutic recommendations remains heterogeneous, with predominance of Level III and IV evidence for most entities, except in the areas of acute hemorrhoids, rectal prolapse, and anal condylomas. Prospective multicenter studies are required to strengthen the evidence base and allow more robust clinical recommendations.
